# *Ganoderma resinaceum* and *Perenniporia fraxinea:* Two Promising Wood Decay Fungi for Pharmaceutical Degradation

**DOI:** 10.3390/jof9050555

**Published:** 2023-05-11

**Authors:** Simone Buratti, Francesca Rinaldi, Enrica Calleri, Marco Bernardi, Desdemona Oliva, Maura Malgaretti, Giuseppe De Girolamo, Barbara Barucco, Carolina Elena Girometta, Elena Savino

**Affiliations:** 1 Department of Earth and Environmental Sciences, University of Pavia, 27100 Pavia, Italy; simone.buratti01@universitadipavia.it (S.B.); carolinaelena.girometta@unipv.it (C.E.G.); elena.savino@unipv.it (E.S.); 2 Department of Drug Sciences, University of Pavia, 27100 Pavia, Italy; enrica.calleri@unipv.it; 3 CAP Holding Spa, Centro Ricerche Salazzurra, Via Circonvallazione Est, 20054 Segrate, Italy; marco.bernardi@gruppocap.it (M.B.); desdemona.oliva@gruppocap.it (D.O.); 4 A2A Ciclo Idrico, Via Lamarmora 230, 25124 Brescia, Italy; maura.malgaretti@a2a.eu (M.M.); giuseppe.degirolamo@a2a.eu (G.D.G.); barbara.barucco@a2a.eu (B.B.)

**Keywords:** myco-remediation, pharmaceuticals, wastewater, diclofenac, paracetamol, ketoprofen

## Abstract

Wood decay fungi (WDF) are a well-known source of enzymes and metabolites which have applications in numerous fields, including myco-remediation. Pharmaceuticals are becoming more problematic as environmental water pollutants due to their widespread use. In this study, *Bjerkandera adusta*, *Ganoderma resinaceum*, *Perenniporia fraxinea*, *Perenniporia meridionalis* and *Trametes gibbosa* were chosen from WDF strains maintained in MicUNIPV (the fungal research collection of the University of Pavia) to test their potential to degrade pharmaceuticals. The degradation potential was tested in spiked culture medium on diclofenac, paracetamol and ketoprofen, three of the most common pharmaceuticals, and irbesartan, a particularly difficult molecule to degrade. *G. resinaceum* and *P. fraxinea* were found to be the most effective at degradation, achieving 38% and 52% (24 h) and 72% and 49% (7 d) degradations of diclofenac, 25% and 73% (24 h) and 100% (7 d) degradations of paracetamol and 19% and 31% (24 h) and 64% and 67% (7 d) degradations of ketoprofen, respectively. Irbesartan was not affected by fungal activity. The two most active fungi, *G. resinaceum* and *P. fraxinea*, were tested in a second experiment in discharge wastewater collected from two different wastewater treatment plants in northern Italy. A high degradation was found in azithromycin, clarithromycin and sulfametoxazole (from 70% up to 100% in 7 days).

## 1. Introduction

Wood decay fungi (WDF) in forest ecosystems play a key role in the degradation of lignocellulosic organic matter, promoting nutrient availability and carbon cycling [1,2]. Based on the type of degradation they perform, WDF can be divided into three main categories: brown rot fungi capable of degrading cellulose and hemicellulose but not lignin, white rot fungi which can degrade all wood components and soft rot fungi that are limited to the degradation of superficial wood [3,4]. The degradation capacity of WDF is enabled by the production of a large spectrum of extracellular enzymes, both ligninolytic and non-ligninolytic, along with other secondary metabolites. Cellulose hydrolysis occurs mainly through exo- and endo-glucanases and cellobiohydrolases; hemicellulose is hydrolyzed mostly by xylanases, glucosidases and mannanases; while lignin is mainly degraded by lignin-peroxidases, manganese peroxidases and laccases [3,5]. Some secondary metabolites also play a key role as cofactors or mediators in the degradation of cell wall components, as enzymes such as peroxidases and laccases are too large to penetrate through cell wall pores. Some metabolites such as veratryl alcohol, oxalate, chlorinated anisyl and chlorinated hydroquinone act as mediators or as substrates in reactions catalyzed by lignocellulosic enzymes or play important roles in the production of other substances that can help the degradation process, such as extracellular H_2_O_2_ production [6,7].

The discovery of the potential for enzymatic and metabolite degradation by WDF has led to the exploitation of fungi and their enzymes in different fields of application [8,9]. Some WDF are well known and have already been used for centuries for their medicinal and nutraceutical properties. Recently, interest has been rising in their potential application in other fields, such as design, cosmetics and textiles, and in the circular economy for sustainable reuse of water resources or waste of various kinds. In the last few years, fungi have been investigated for use in the production of new materials, so called myco-materials [10], recycling of agro-industrial wastes [11,12] and particularly for enzyme-centered degradation of pollutants such as plastics, hydrocarbons, pesticides, dyes and pharmaceuticals in soil and water [9,13,14].

Some pollutants have become more problematic with the recent COVID-19 pandemic due to the large increase in single-use plastics, such as masks and gloves, and the increased consumption of pharmaceuticals and antibiotics [15]. Pharmaceuticals and their metabolites enter wastewater treatment plants where they are not completely eliminated, and the residual concentration is discharged in superficial and ground water [16,17,18]. Nowadays, the European Union specifies quality standards and priority contaminants but there are no concentration limits for pharmaceuticals in discharge waters [19]. A watch-list exists, which is updated every two years, to monitor emerging contaminants and their possible effects on the environment and human health [20].

WDF have the potential to simultaneously reduce pharmaceuticals and other pollutants in water in a sustainable way thanks to their production of a variety of enzymes. Some species of fungi such as *Tramtes versicolor* (L.) Lloyd, the most used and researched species, *Pleurotus ostreatus* (Jacq.) P. Kumm. and *Phanerochaete chrysosporium* Burds. have been widely studied and have proven to be effective on some pharmaceutical compounds such as diclofenac, ketoprofen and ibuprofen [21,22,23,24,25,26]. Among WDF, many species are yet to be studied and may have the capability to degrade pharmaceuticals and other contaminants. It is important to investigate their potential to further extend the range of applications for myco-remediation.

Many studies have identified *Bjerkandera adusta* (Willd.) as a promising species for remediation/degradation applications [27,28,29]. In addition, *Perenniporia meridionalis* (Decock and Stalpers) has already been shown to have good Mn peroxidase activity [30], and *Trametes gibbosa* (Pers.) Fr. could prove to be of interest if it is found to possess similar capabilities as *T. versicolor*. In nature, *Ganoderma resinaceum* Boud. and *Perenniporia fraxinea* (Bull.) Ryvarden are plant saprotrophs and pathogens capable of easily degrading wood matter and both are aggressive towards the host [31,32]. This ability could be translated into a strong enzymatic action that could be exploited for myco-remediation, bringing to light interesting new results. *P. fraxinea* is also a relatively poorly studied species and its qualities and possible applications have only recently been discovered. *P. fraxinea* has been used for carotenoid extraction and it has been established that this species can produce enzymes with fibrinolytic properties that can be used in medicine against thrombosis. Moreover, certain strains of *P. fraxinea* have shown the ability to degrade some dyes and to tolerate and accumulate heavy metals such as Cd, Hg and Cu [33,34,35,36].

The Fungal Research Culture Collection MicUNIPV of the University of Pavia (Italy) maintains 600 fungal strains belonging to 130 different species, and many of these strains still have unexplored potential.

The aim of this study is to provide an indication of which WDF species may be promising for future myco-remediation applied studies or for exploitation of enzymes.

The first experiment investigated the pharmaceutical degradation potential of *B. adusta*, *G. resinaceum, P. fraxinea*, *P. meridionalis* and *T. gibbosa* in a sterile liquid culture medium spiked with diclofenac, paracetamol and ketoprofen, three of the most common pharmaceuticals found in wastewaters, and irbesartan, a molecule that is difficult degrade in conventional wastewater treatment methods. Irbesartan is an orally active lipophilic pharmaceutical used to treat hypertension and diabetic nephropathy, characterized by a high permeability and low solubility and is commonly detected both in groundwater and in drinking water. Irbesartan is also a molecule that remains stable when subjected to photodegradation and to hydrolysis and oxidation under certain conditions. A number of experiments on the degradability of sartans have also shown that irbesartan has one of the lowest degradation percentages [37,38]. The second experiment investigated the degradation potential of the two best strains previously identified in wastewater discharged from two different wastewater treatment plants (WWTPs).

## 2. Materials and Methods

### 2.1. Choice of Wood Decay Fungi (WDF)

The *Bjerkandera adusta*, *Perenniporia fraxinea*, *Perenniporia meridionalis* and *Trametes gibbosa* strains belonging to the Fungal Research Culture Collection MicUNIPV of Department of Earth and Environmental Sciences, University of Pavia (Italy), and a strain of *Ganoderma resinaceum* belonging to MOGU Srl (MOGU’s Fungal Strain Collection—MFSC) were chosen based on a literature review.

All the strains were previously obtained from basidiomata collected in northern Italy, isolated in pure culture, identified by ITS rDNA analyses as reported by Cartabia et al. 2022 [39] and maintained in 2% malt extract agar medium (MEA, Biokar diagnostics, Allonne, France and VWR Chemicals, Milano, Italy) at 3 °C.

### 2.2. Pharmaceuticals and Discharge Wastewater

Diclofenac sodium DCF (CAS: 15307-79-6), irbesartan IRS (CAS: 138402-11-6), ketoprofen KET (CAS: 22071-15-4) and paracetamol PCT (Acetaminophen CAS: 103-90-2) were all purchased from Sigma-Aldrich (St. Louis, MO, USA).

Discharge wastewater was taken at the end of water line in two urban wastewater treatment plants in Lombardy, northern Italy, hereinafter referred to as WWTP1 and WWTP2.

The pharmaceuticals tested in discharged wastewater are listed in Table 1. All discharged wastewater samples were analysed by Eurolab Analysis and Results Srl.

### 2.3. Experimental Procedure in Liquid Culture Medium

For each fungal strain, 500 mL flasks containing 200 mL of 2% malt extract (ME) liquid medium were prepared by inoculating agar plugs (surface of about 1 cm^2^) from plates with active growing mycelium. Flasks were incubated for 10 days at 120 rpm at 25 °C. Mycelium grew in agitation conditions with spherical structures, referred to as pellets. The content of each flask was drained and the pellets were washed with sterile distilled water then gently squeezed to remove excess water.

For each strain, four 100 mL flasks containing 70 mL of ME 2% were prepared (one for each pharmaceutical to be tested). In each of the four flasks, only one pharmaceutical was spiked to obtain the desired concentration of 10 μg/L and then flasks were inoculated with 1 g (wet weight) of pellets.

A control flask for each pharmaceutical was prepared containing ME 2% and the 10 μg/L solution of the compound in order to evaluate the pharmaceuticals’ natural degradation.

Each experiment was performed in triplicate and flasks were incubated in the dark at 25 °C in static (unstirred) conditions.

An aliquot of liquid medium was taken from each flask after 24 h and after 7 days to analyse the pharmaceutical concentration.

To ensure that the variation in drug concentration was not due to pharmaceutical adsorption on the fungal cell wall, parallel tests with dead mycelium were carried out. Agar plugs with active growing mycelium were inoculated in 100 mL flasks with 70 mL of ME 2% and incubated for 7 days. At the end of incubation, flasks were autoclaved at 120 °C for 30 min to kill the fungal biomass. The flasks were allowed to cool to room temperature and then 10 μg/L of pharmaceutical was added.

### 2.4. Analytical Procedures

Samples were prepared by ultracentrifugation of 1 mL of each medium solution at 14,000 rpm for 5 min to remove fungal residues. The derived supernatants were analysed by high performance liquid chromatography (HPLC) coupled to mass spectrometry (MS).

Analyses were performed by an ExionLC system equipped with a degasser, a quaternary pump, an autosampler and a thermostated column compartment (SCIEX, Framingham, MA, USA) and a X500QTOF mass spectrometer (SCIEX, Framingham, MA, USA) with an ESI source. The LC-MS system was controlled by SCIEX OS software (1.7 version).

Diclofenac samples were analysed using an Eclipse XDB-C18 column (5.0 µm, 4.6 × 150 mm, Agilent Technologies, Santa Clara, CA, USA), while for irbesartan, ketoprofen and paracetamol, a XTerra^®^ MS C18 column (5.0 µm, 2.1 × 250 mm, Waters, Milford, MA, USA) was used.

A column temperature of 25 °C, an injection volume of 20 µL and isocratic elution were applied to all samples.

The mobile phases were composed of 0.1% formic acid in water/acetonitrile (50:50, *v*/*v*) for diclofenac or 0.1% formic acid in water/0.1% formic acid in acetonitrile for the other compounds (65:35, *v*/*v* for irbesartan and ketoprofen and 97:3, *v*/*v* for paracetamol).

The flow rate was set at 1 mL/min for diclofenac samples and 0.3 mL/min for the other pharmaceuticals.

The following MS parameters were applied: curtain gas, 30 psi; ion source gas 1, 40 psi; ion source gas 2, 50 psi (diclofenac) or 45 psi (irbesartan, ketoprofen and paracetamol); temperature, 450 °C (diclofenac) or 350 °C (irbesartan, ketoprofen and paracetamol); polarity, positive; ion spray voltage, 5500 V; CAD gas, 7; TOF start mass, 100 Da; TOF stop mass, 1000 Da; accumulation time, 1 s; declustering potential, 60 V; and collision energy, 10 V.

The pharmaceutical degradation percentages were calculated as follows:

$$100-\frac{\mathrm{pharmaceutical}\;\mathrm{area}\;\mathrm{in}\;\mathrm{the}\;\mathrm{sample}\;}{\mathrm{average}\;\mathrm{pharmaceutical}\;\mathrm{area}\;\mathrm{in}\;\mathrm{the}\;3\;\mathrm{control}\;\mathrm{samples}}\times 100$$

For each analyte, the limit of detection (LOD) was calculated from the signal/noise (S/N) ratios of samples at different concentrations, normalized for their concentrations (C) as follows:

$$\mathrm{LOD}=C\;\frac{3}{S/N}$$

where S/N = 2H/h, as defined by USP [41]. The reported LODs were calculated as average values of six analyses for each analyte. The LOD was 1.36 μg/L for diclofenac, 0.22 μg/L for irbesartan, 0.79 μg/L for ketoprofen and 1.72 μg/L for paracetamol.

### 2.5. Experimental Procedure in Wastewater

The two best performing strains from the experiment in sterile liquid culture medium were tested in discharge wastewater, a clear water matrix with a small and variable quantity of solid particulates. For each fungal strain, flasks for pellet formation were prepared as described in Section 2.3.

For each strain, four 1 L flasks with 400 mL of discharge wastewater were prepared, one for each WWTP and one for each sampling time (24 h and 7 days). Each flask was inoculated with 5 g (wet weight) of pellets.

A control flask for each WWTP and sampling time was prepared containing only discharge wastewater. A sample was also taken at the moment of water collection directly from the treatment plant (T0 sample).

Flasks were incubated in the dark at 25 °C in static conditions.

A 50 mL aliquot of liquid medium was taken from each flask after 24 h and after 7 days and analysed by Eurolab Analysis and Results Srl with LC-MS/MS to determine the pharmaceuticals’ concentration.

## 3. Results

### 3.1. Sterile Liquid Culture Medium

**Figure 1:**
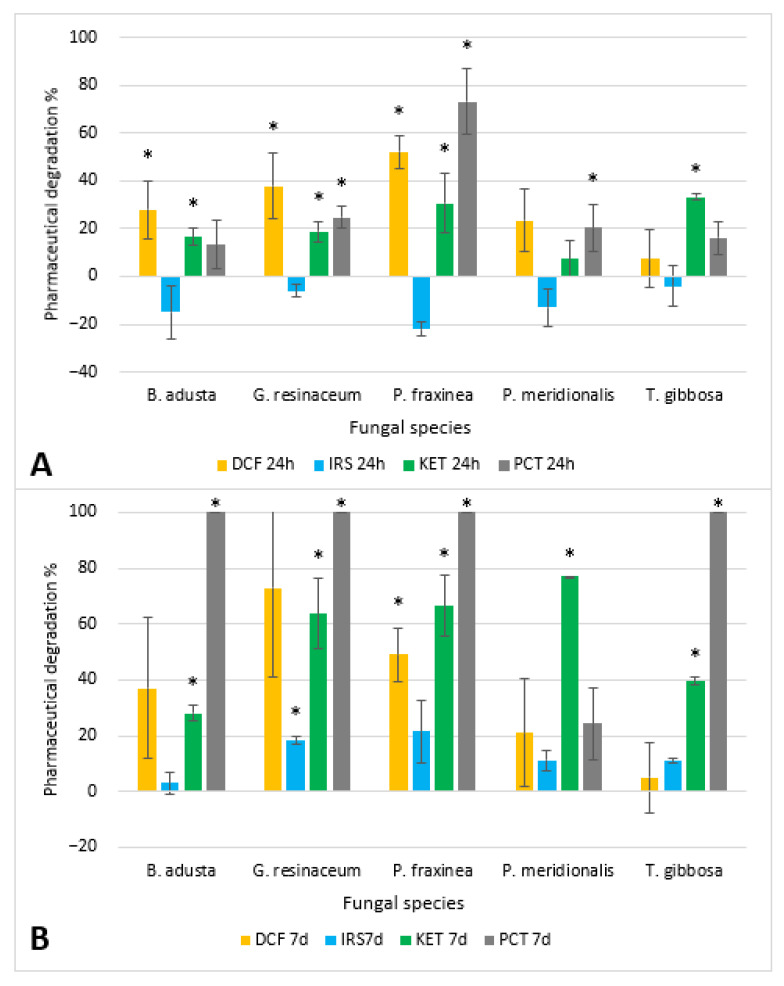
Pharmaceutical degradation percentages after 24 h (**A**) and after 7 days (**B**) for each strain tested. Significantly different concentrations compared to the control are marked by * based on a *t*-test (* *p*-value < 0.05).

The degradation results obtained from each strain are reported in Figure 1.

Diclofenac was reduced by up to 50% in 24 h by *P. fraxinea* and more than 50% by *G. resinaceum* and *P. fraxinea* in 7 days. No strain was able to decrease Irbesartan more than 30% even after 7 days. Ketoprofen was degraded in the range of 10–30% in 24 h and more than 60% by 3/5 strains in 7 days. Only one strain, *P. fraxinea,* was capable of halving the paracetamol concentration in 24 h, while after 7 days 4/5 strains achieved its complete degradation.

The results obtained from samples inoculated with dead mycelium showed similar concentrations as the malt extract control, thus excluding the adsorption component from pharmaceutical degradation.

#### 3.1.1. Diclofenac

*P. fraxinea* and *G. resinaceum* reported higher diclofenac degradation percentages (52% and 38%, respectively) in the first 24 h compared to other strains; *T. gibbosa* had the lowest degradation capabilities (5–7%). *G. resinaceum* seemed to achieve an even greater diclofenac degradation after 7 days, but the standard deviation was too high to consider this result reliable. *P. fraxinea* was the only strain where the diclofenac concentration, after fungal activity, was statistically different from the malt extract control (*p* value < 0.05), both after 24 h and after 7 days. *B. adusta* and *G. resinaceum* had a statistically significant effect only in the 24 h samples due to the high standard deviation of the 7 day sample. In all strains tested, there were no statistically significant differences in concentrations between 24 h and 7 days.

#### 3.1.2. Irbesartan

Irbesartan exhibited a very low degradation rate compared to the other pharmaceuticals tested, with an apparent increase in pharmaceutical concentrations at 24 h. The highest degradation percentage was obtained by the use of *P.fraxinea* (22%), followed by *G. resinaceum* (18%), even if the former had a high variability compared to the latter. *G. resinaceum* is also the only strain that had a statistically different irbesartan concentration compared to control, though after 7 days.

There was a statistically significant difference between concentration registered after 24 h and 7 days in *G. resinaceum*, *P. fraxinea* and *P. meridionalis*.

#### 3.1.3. Ketoprofen

The strains that exhibited a high ketoprofen degradation after 24 h were *T. gibbosa* (33%) and *P. fraxinea* (31%). After 7 days of incubation, the highest degradation was achieved by *P. meridionalis* (77%), followed by *P. fraxinea* (66%) and *G. resinaceum* (64%). The strain with the overall lowest degradation was *B. adusta*.

The concentration of ketoprofen was statistically different in samples treated with *B. adusta*, *G. resinaceum*, *P. fraxinea* and *T. gibbosa*, at both incubation times. In *P. meridionalis* samples, the difference was significant only after 7 days.

In all samples except *T. gibbosa*, a 7 day incubation time achieved higher degradation percentages than those achieved after 24 h, with statistically different values from the control.

#### 3.1.4. Paracetamol

Paracetamol degradation tests yielded better long-term results compared to other pharmaceuticals. Degradation at 24 h was achieved with optimal results only by *P. fraxinea* (73%), while at 7 days, all strains, except *P. meridionalis*, achieved almost complete degradation, lowering the paracetamol concentration below the analytical LOD. Degradation percentages were statistically different from the control at 24 h in *G. resinaceum*, *P. fraxinea* and *P. meridionalis* and at 7 days in *B. adusta*, *G. resinaceum*, *P. fraxinea* and *T. gibbosa*.

Concentrations between 24 h and 7 day samples were statistically different only in *B. adusta*, *G. resinaceum* and *T. gibbosa*.

### 3.2. Discharge Wastewater

The two best performing strains from the sterile liquid culture medium experiment were *G. resinaceum* and *P. fraxinea*. They were tested in discharge wastewater to see how the degradation performance obtained in sterile conditions could be altered in a real water matrix containing a mixture of pharmaceuticals and other organisms.

The concentration of pharmaceuticals in wastewater is never constant, but rather subjected to variations related to multiple factors such as the season. Table 2 shows the ranges of concentrations of compounds recorded in the two WWTPs examined.

**Figure 2:**
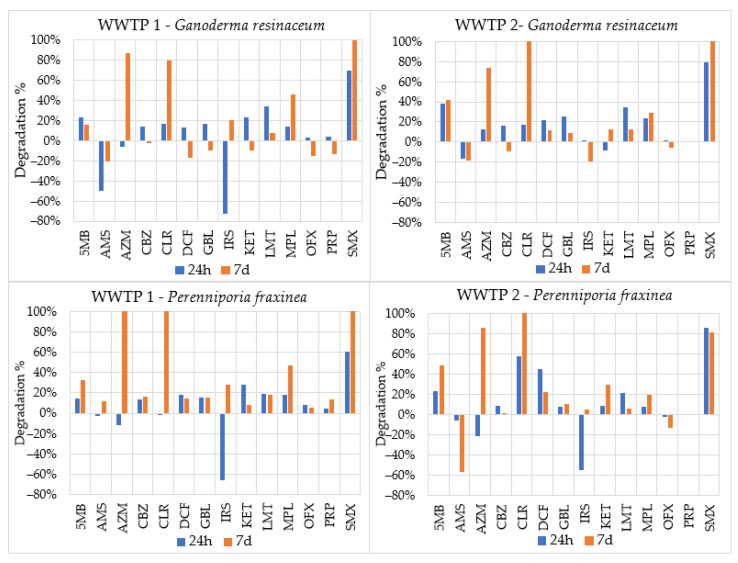
Pharmaceutical and molecule degradation percentages after 24 h and 7 days from fungal inoculum compared to the T0 control.

The percentages of degraded molecules compared to the T0 control for each strain and each WWTP are reported in Figure 2. The degradation percentages by fungal activity are reported net of degradation occurring in the control sample. The T0 control is a non-fungal inoculated sample, chosen to verify the normal degradation of compounds due to other biotic or abiotic factors.

The pharmaceuticals most affected by degradative activity were AZM, CLR and SMX, with noticeable effects (degradation percentages between 73% and 100%) only after 7 days, except for SFX whose degradation was between 61% and 85% after 24 h. Negative degradation—an increase in the concentration of the compound—was registered for some pharmaceuticals, with AMS and IRS being affected the most (−57–−71%).

*G. resinaceum* achieved better degradation results compared to *P. fraxinea* in 24 h for 7/14 molecules (PRP values are the same in both strains) in WWTP1 wastewater and 8/14 molecules (PRP values are the same in both strains) in WWTP2 wastewater. On the contrary, *P. fraxinea* achieved higher degradation percentages after 7 days for 13/14 compounds (SMX values are the same in both strains) in WWTP1 wastewater and 7/14 compounds (CLR and PRP values are the same in both strains) in WWTP2 wastewater. *G. resinaceum* obtained, on average, higher degradation values in discharge wastewater from WWTP2, while *P. fraxinea* had higher degradation in discharge wastewater from WWTP1.

DCF, IRS and KET achieved comparable values to those obtained in sterile liquid culture medium experiment only in certain cases:

- DCF: after 24 h in WWTP2 discharge wastewater inoculated with *P. fraxinea*;
- IRS: after 24 h in WWTP1 discharge wastewater for both strains;
- KET: after 7 days in WWTP1 discharge wastewater for both strains.

In all the other cases, the degradation percentages in wastewater were lower compared to the liquid culture medium.

## 4. Discussion

The results obtained indicated that some of the tested species possess potential for degradation, as they were capable of breaking down pharmaceuticals even in a short time period.

Diclofenac appears to be the easiest degraded pharmaceutical in a short-term incubation, while paracetamol was the most easily degraded over long periods.

The ease of degradation by fungi likely depends on their enzymes and the type of bonds the target molecule possesses. Molecules with electron donating groups, such as amine and hydroxyl groups, are reported to be removed more efficiently by white rot fungi, while electron withdrawing functional groups, such as carboxylic, chlorine and amide groups, makes the molecule less oxidizable and thus more difficult to degrade [42]. The chemical structures of the pharmaceuticals and molecules analyzed are reported in Table 3.

In the liquid culture medium experiment, the fact that paracetamol has both a hydroxyl and a secondary amine group explains the high degradation percentages obtained [25,42]. Complete degradation of paracetamol was also achieved in other studies with *T. versicolor* in the same time span [23,26].

On the contrary, ketoprofen contains a carboxyl group, while irbesartan has an amide group. Their chemical structure could be the reason why the tested strains were capable of degrading ketoprofen only after 7 days, while they were not able to substantially lower the irbesartan concentration even after longer periods. Irbesartan also showed an apparent increase (negative degradation percentage), probably due to fungal metabolites that could alter the pH of the solution. This might affect the solubility and ionization of irbesartan and have an impact on detection in mass spectrometry. This compound is also characterized by a much lower solubility in water compared to the other tested pharmaceuticals and it therefore might be more affected by the matrix effect. Ketoprofen needs a longer contact time to achieve a good degradation and this is also confirmed by other studies, in which *Trametes versicolor* showed a high degradation (80%) of ketoprofen after 21 days, while *P. ostreatus* degraded it by 36% in 2 days [22,25,27,43].

For diclofenac, a high degradation achieved in 24 h was also confirmed by numerous studies that report its highly efficient removal by fungi [22,25,44]. The diclofenac degradation in our samples was lower compared to other works, where synthetic or defined liquid media treated with WDF *Trametes versicolor*, *Pleurotus ostreatus* or *Bjerkandera adusta* showed a very high degradation (80–99%) of diclofenac in a time range between 1 and 7 days.

Among strains tested in liquid culture media, *P. fraxinea* was the species with the greatest myco-remediation potential, followed by *G. resinaceum.* The mycelium of these two fungi achieved, in almost every experiment, the highest degradation percentage compared to other species. Even though both species were found to be excellent degraders, *P. fraxinea* is preferable for application due to the reduced variability of the results obtained compared to those of *G. resinaceum*. Compared to other highly performing fungal strains largely tested in the literature, such as the aforementioned *T. versicolor*, these two strains reach higher degradation percentages in the first 24 h for ketoprofen and paracetamol, acting faster compared to other known strains. On the contrary, their enzymatic action on diclofenac does not achieve the high degradation rate found in the literature.

Irbesartan still remains an understudied molecule regarding myco-remediation and there is insufficient data to make a comparison.

Of particular interest is that *P. meridionalis*, although not performing well in general, had specific degradative activity for ketoprofen, reaching 77% of degradation after 7 days. Further studies are needed to understand the specificity of its enzymes towards ketoprofen.

The good degradation of diclofenac and ketoprofen obtained in liquid culture media was not achieved in discharge wastewater, where degradation was overall low. This could be explained by enzyme activity focusing more on easily degradable molecules such as, in this case, azithromycin, clarithromycin and sulfamethoxazole. Azithromycin and clarithromycin are two molecules with a high number of electron-donating groups; the former has five hydroxyl groups and two tertiary amine groups and the latter has four hydroxyl groups and a tertiary amine group. Sulfamethoxazole is reported to reach generally high levels of degradation. Diclofenac removal is usually efficient but highly variable, ranging from a few hours to a few days depending on the fungal species used and enzyme type [42].

For some pharmaceuticals, in particular those degraded by *G. resinaceum*, such as CBZ, DCF, GBL, KET, OFX and PRP, after an initial degradation of the compound, an increase in concentration (negative degradation percentages) was found in 7 day samples. This phenomenon could be explained with desorption and/or deconjugation processes. Desorption involves the liberation of pharmaceuticals and other compounds from suspended solids and particles present in the water, often due to microorganism activity. Deconjugation concerns the metabolization of pharmaceuticals in the human body that transforms them into better absorbing compounds. In the end, both the parent and metabolized (conjugated) pharmaceuticals are excreted from the body and can be found in wastewaters. These metabolites could undergo deconjugation processes that transform them back to the parent compound, thus leading to an increase in concentration [26,45,46,47,48]. In this case, enzymes appear to act first on pharmaceuticals already present in wastewater and then to desorb those in the suspended solids. Desorption of pharmaceuticals from suspended solids and deconjugation phenomena are also present in the control sample without fungal inoculum, but their effect is limited when compared with inoculated samples in which the degradative action on the suspended solids by enzyme activity and also by mechanical action of hyphae is more pronounced.

The opposite effect was registered with amisulpride and irbesartan, where an initial increase in concentration, probably due to the above-mentioned phenomena, was followed by a decrease over the 7 day period. In this case, because these two molecules are difficult to degrade, enzymes seem to first act on the particulates, releasing desorbed pharmaceuticals and causing an increase in their concentration, and only then degrading them.

The overall low degradation performances compared to culture liquid medium are probably also due to the discharge water, which consists of a relatively clean matrix containing a few nutrients that the fungus can use for metabolism and enzyme production. Furthermore, several studies report that substances commonly found in wastewater, such as NaCl, sulfides, halides, organic compounds and heavy metals, can alter or partially inhibit the activity of some enzymes. Competition for nutrients with bacteria and other microorganisms can slow fungal growth and reduce its efficiency to produce enzymes, thus decreasing the degradation of pollutants [23,49,50].

Compared to other microorganisms often used for bioremediation of waters such as algae, *P. fraxinea* shows the potential to achieve a higher degradation of diclofenac if we compare the 45% degradation achieved in WWTP2 in 24 h to the 22–79% in 5–9 days achieved by some algae species [51]. *G. resinaceum* and *P. fraxinea* achieve higher clarithromycin degradation (80–100%) in 24 h, whereas algae achieve the same in 7 days. Metroprolol degradation by these two fungi is lower (47%) compared to some algae species that could reach 99% removal. Sulfamethoxazole is a pharmaceutical almost completely degraded (99–100%) by *G. resinaceum* and *P. fraxinea*, while degradation by algae varies between 46% and 100% [51,52]. Carbamazepine is an example of a pharmaceutical of which both these fungal strains and algae struggle to degrade, with a maximum of 16% for *P. fraxinea* and 14–30% for algae [53].

## 5. Conclusions

WDF are proven to be of value in the degradation of emerging contaminants. Among the strains chosen and tested, *Perenniporia fraxinea* and *Ganoderma resinaceum* were found to be able to lower the concentrations of diclofenac, ketoprofen and paracetamol with high degradation percentages. *P. fraxinea* is the most promising strain, with a 52% diclofenac degradation (24 h), a 67% ketoprofen degradation (7 days) and a 73% paracetamol degradation in 24 h, increasing to 100% after 7 days.

The poor degradability of irbesartan, probably due to the compound’s chemical structure and functional groups, was reconfirmed in this study. Indeed, only *G. resinaceum* was able to achieve a low but significant reduction.

In discharge wastewater, even though some pharmaceuticals remain tough to remove, the enzymatic activities of *G. resinaceum* and *P. fraxinea* could still be exploited to degrade AZM, CLR and SMX (from 70% up to 100%).

This study identified two species, relatively new to this field of mycology, that can be exploited for various applications. Myco-remediation of pharmaceuticals in water and wastewaters can be problematic due to the strong pathogenic nature of the two strains identified. Therefore, their exploitation for remediation purposes should be performed in a way that limits spores and propagule propagation in the environment, for example, through mycelium or enzyme immobilization techniques.

Further studies are needed to understand (1) the best contact time to optimize the pharmaceutical degradation between 24 h and 7 days, (2) the strategies that could be implemented to reduce the problem related to their pathogenicity and (3) which growth conditions can improve their degradative performance.

## Figures and Tables

**Table 1:** Pharmaceuticals and molecules tested in discharged wastewater by Eurolab Analysis and Results Srl. Abbreviations used in this study and classification and CAS numbers for each compound are reported (Pubchem.com, accessed on 14 January 2023) [40].

Name	Abbreviation	Classification	CAS Number
5 Methyl-Benzotriazole	5 MB	Corrosion inhibitor and ultraviolet light inhibitor, used for aircraft de-icing agents, plastic stabilizers, anti-fogging agents, pharmaceuticals, fungicides, paints and coatings	136-85-6
Amisulpride	AMS	Antipsychotic and antidepressive agent	71675-85-9
Azithromycin	AZM	Antibiotic	83905-01-5
Carbamazepine	CBZ	Anticonvulsant and analgesic	298-46-4
Clarithromycin	CLR	Antibiotic	81103-11-9
Diclofenac	DCF	Non-steroidal anti-inflammatory agent with antipyretic and analgesic actions	15307-86-5
Gabapentin-Lactam	GBL	Transformation product of gabapentin (anti-epileptic)	64744-50-9
Irbesartan	IRS	Nonpeptide angiotensin II antagonist with antihypertensive activity	138402-11-6
Ketoprofen	KET	Anti-inflammatory analgesic and antipyretic	22071-15-4
Lamotrigine	LMT	Antiepileptic and analgesic	84057-84-1
Metoprolol	MPL	Beta-adrenergic antagonist and antihypertensive	51384-51-1
Ofloxacin	OFX	Antibiotic	82419-36-1
Propyphenazone	PRP	Non-steroidal anti-inflammatory and non-narcotic analgesic	479-92-5
Sulfamethoxazole	SMX	Antibiotic	723-46-6

**Table 2:** Pharmaceuticals and molecules tested, with minimum and maximum concentrations found in the discharge water of WWTPs under study.

Molecule	WWTP 1 Concentration Range (ng/L) (Min–Max)	WWTP 2 Concentration Range (ng/L) (Min–Max)
5 Methyl–Benzotriazole	3228–13,089	331–1159
Amisulpride	28–36	33–130
Azithromycin	254–458	120–277
Carbamazepine	197–362	130–325
Clarithromycin	32–227	29–90
Diclofenac	198–2914	224–898
Gabapentin–Lactam	54–118	267–465
Irbesartan	223–638	55–240
Ketoprofen	14–199	22–112
Lamotrigine	80–186	143–320
Metoprolol	63–83	32–82
Ofloxacin	124–204	23–190
Propyphenazone	<10–22	<10–11
Sulfamethoxazole	<10–226	38–176

**Table 3:** Name and chemical structures of the tested compounds (Pubchem.com).

Name	Chemical Structure	Name	Chemical Structure
5 Methyl-Benzotriazole	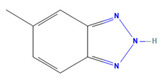	Amisulpride	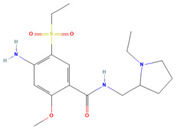
Azithromycin	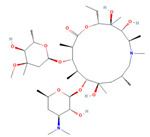	Carbamazepine	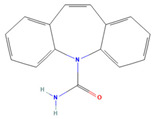
Clarithromycin	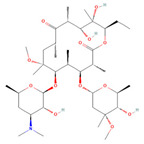	Diclofenac	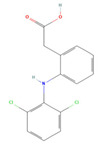
Gabapentin-Lactam	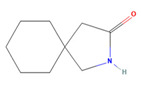	Irbesartan	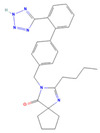
Ketoprofen	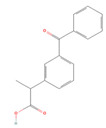	Lamotrigine	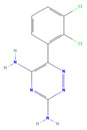
Metoprolol	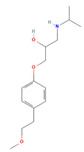	Ofloxacin	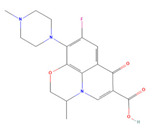
Paracetamol	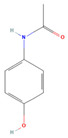	Propyphenazone	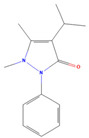
Sulfamethoxazole	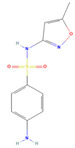		

## Data Availability

The data presented in this study are contained within the article.

## References

[1] Lonsdale D., Pautasso M., Holdenrieder O. (2008). Wood-Decaying Fungi in the Forest: Conservation Needs and Management Options. Eur. J. For. Res..

[2] Mäki M., Mali T., Hellén H., Heinonsalo J., Lundell T., Bäck J. (2021). Deadwood Substrate and Species-Species Interactions Determine the Release of Volatile Organic Compounds by Wood-Decaying Fungi. Fungal Ecol..

[3] Bucher V.V.C., Pointing S.B., Hyde K.D., Reddy C.A. (2004). Production of Wood Decay Enzymes, Loss of Mass, and Lignin Solubilization in Wood by Diverse Tropical Freshwater Fungi. Microb. Ecol..

[4] Schwarze F.W.M.R. (2007). Wood Decay under the Microscope. Fungal Biol. Rev..

[5] van den Brink J., de Vries R.P. (2011). Fungal Enzyme Sets for Plant Polysaccharide Degradation. Appl. Microbiol. Biotechnol..

[6] Schick Zapanta L., Tien M. (1997). The Roles of Veratryl Alcohol and Oxalate in Fungal Lignin Degradation. J. Biotechnol..

[7] Ten Have R., Teunissen P.J.M. (2001). Oxidative Mechanisms Involved in Lignin Degradation by White-Rot Fungi. Chem. Rev..

[8] Covino S., Stella T., Cajthaml T., Purchase D. (2016). Mycoremediation of Organic Pollutants: Principles, Opportunities, and Pitfalls. Fungal Applications in Sustainable Environmental Biotechnology.

[9] Hyde K.D., Xu J., Rapior S., Jeewon R., Lumyong S., Niego A.G.T., Abeywickrama P.D., Aluthmuhandiram J.V.S., Brahamanage R.S., Brooks S. (2019). The Amazing Potential of Fungi: 50 Ways We Can Exploit Fungi Industrially. Fungal Divers..

[10] Cartabia M., Girometta C.E., Milanese C., Baiguera R.M., Buratti S., Branciforti D.S., Vadivel D., Girella A., Babbini S., Savino E. (2021). Collection and Characterization of Wood Decay Fungal Strains for Developing Pure Mycelium Mats. J. Fungi.

[11] Israilides C., Philippoussis A. (2003). Bio-Technologies of Recycling Agro-Industrial Wastes for the Production of Commercially Important Fungal Polysaccharides and Mushrooms. Biotechnol. Genet. Eng. Rev..

[12] Sadh P.K., Duhan S., Duhan J.S. (2018). Agro-Industrial Wastes and Their Utilization Using Solid State Fermentation: A Review. Bioresour. Bioprocess..

[13] Noman E., Al-Gheethi A., Mohamed R.M.S.R., Talip B.A. (2019). Myco-Remediation of Xenobiotic Organic Compounds for a Sustainable Environment: A Critical Review. Top. Curr. Chem..

[14] Akhtar N., Mannan M.A. (2020). Mycoremediation: Expunging Environmental Pollutants. Biotechnol. Rep..

[15] Rizvi S.G., Ahammad S.Z. (2022). COVID-19 and Antimicrobial Resistance: A Cross-Study. Sci. Total Environ..

[16] Bottoni P., Caroli S., Caracciolo A.B. (2010). Pharmaceuticals as Priority Water Contaminants. Toxicol. Environ. Chem..

[17] Quesada H.B., Baptista A.T.A., Cusioli L.F., Seibert D., de Oliveira Bezerra C., Bergamasco R. (2019). Surface Water Pollution by Pharmaceuticals and an Alternative of Removal by Low-Cost Adsorbents: A Review. Chemosphere.

[18] O’Flynn D., Lawler J., Yusuf A., Parle-McDermott A., Harold D., Mc Cloughlin T., Holland L., Regan F., White B. (2021). A Review of Pharmaceutical Occurrence and Pathways in the Aquatic Environment in the Context of a Changing Climate and the COVID-19 Pandemic. Anal. Methods.

[19] Directive 2013/39/EU of the European Parliament and of the Council of 12 August 2013 Amending Directives 2000/60/EC and 2008/105/EC as Regards Priority Substances in the Field of Water. PolicyText with EEA Relevance. https://eur-lex.europa.eu/eli/dir/2013/39/oj.

[20] Giardina S., Castiglioni S., Corno G., Fanelli R., Maggi C., Migliore L., Sabbatucci M., Sesta G., Zaghi C., Zuccato E. (2021). Approccio Ambientale All’antimicrobico-Resistenza.

[21] Marco-Urrea E., Pérez-Trujillo M., Cruz-Morató C., Caminal G., Vicent T. (2010). White-Rot Fungus-Mediated Degradation of the Analgesic Ketoprofen and Identification of Intermediates by HPLC–DAD–MS and NMR. Chemosphere.

[22] Marco-Urrea E., Pérez-Trujillo M., Cruz-Morató C., Caminal G., Vicent T. (2010). Degradation of the Drug Sodium Diclofenac by Trametes versicolor Pellets and Identification of Some Intermediates by NMR. J. Hazard. Mater..

[23] Asif M.B., Hai F.I., Singh L., Price W.E., Nghiem L.D. (2017). Degradation of Pharmaceuticals and Personal Care Products by White-Rot Fungi—A Critical Review. Curr. Pollut. Rep..

[24] Mir-Tutusaus J.A., Baccar R., Caminal G., Sarrà M. (2018). Can White-Rot Fungi Be a Real Wastewater Treatment Alternative for Organic Micropollutants Removal? A Review. Water Res..

[25] Dalecka B., Juhna T., Rajarao G.K. (2020). Constructive Use of Filamentous Fungi to Remove Pharmaceutical Substances from Wastewater. J. Water Process Eng..

[26] Tormo-Budowski R., Cambronero-Heinrichs J.C., Durán J.E., Masís-Mora M., Ramírez-Morales D., Quirós-Fournier J.P., Rodríguez-Rodríguez C.E. (2021). Removal of Pharmaceuticals and Ecotoxicological Changes in Wastewater Using Trametes versicolor: A Comparison of Fungal Stirred Tank and Trickle-Bed Bioreactors. Chem. Eng. J..

[27] Rodarte-Morales A.I., Feijoo G., Moreira M.T., Lema J.M. (2011). Degradation of Selected Pharmaceutical and Personal Care Products (PPCPs) by White-Rot Fungi. World J. Microbiol. Biotechnol..

[28] Gupta N., Tripathi A.K., Harsh N.S.K. (2011). Bioremediation of Cotton-Textile Effluent Using Fungi. BEPLS Bull. Environ. Pharmacol. Life Sci..

[29] Dhiman N., Jasrotia T., Sharma P., Negi S., Chaudhary S., Kumar R., Mahnashi M.H., Umar A., Kumar R. (2020). Immobilization Interaction between Xenobiotic and Bjerkandera adusta for the Biodegradation of Atrazine. Chemosphere.

[30] Doria E., Altobelli E., Girometta C., Nielsen E., Zhang T., Savino E. (2014). Evaluation of Lignocellulolytic Activities of Ten Fungal Species Able to Degrade Poplar Wood. Int. Biodeterior. Biodegrad..

[31] Sillo F., Savino E., Giordano L., Girometta C., Astegiano D., Picco A.M., Gonthier P. (2016). Analysis of genotypic diversity provides a first glimpse on the patterns of spread of the wood decay fungus Perenniporla fraxinea in an urban park in northern Italy. J. Plant Pathol..

[32] Bernicchia A., Gorjon S.P. (2020). Polypores of the Mediterranean Region.

[33] Arbind K., Jagdeep K. (2011). Fibrinolytic Agents in Reference to Fungi: An Overview. J. Pharm. Res..

[34] Choi Y.-S., Seo J.-Y., Lee H., Yoo J., Jung J., Kim J.-J., Kim G.-H. (2014). Decolorization and Detoxification of Wastewater Containing Industrial Dyes by Bjerkandera adusta KUC9065. Water Air Soil Pollut..

[35] Sturini M., Girometta C., Maraschi F., Savino E., Profumo A. (2017). A Preliminary Investigation on Metal Bioaccumulation by Perenniporia fraxinea. Bull. Environ. Contam. Toxicol..

[36] Kim J., Lee J.H. (2020). Development of carotenoid production process using Perenniporia fraxinea. J. Mushroom.

[37] Husain A., Md Mitra S.A.M., Bhasin P.S. (2011). A review of pharmacological and pharmaceutical profile of irbesartan. Pharmacophore.

[38] Ladhari A., La Mura G., Di Marino C., Di Fabio G., Zarrelli A. (2021). Sartans: What They Are for, How They Degrade, Where They Are Found and How They Transform. Sustain. Chem. Pharm..

[39] Cartabia M., Girometta C.E., Baiguera R.M., Buratti S., Babbini S., Bernicchia A., Savino E. (2022). Lignicolous Fungi Collected in Northern Italy: Identification and Morphological Description of Isolates. Diversity.

[40] PubChem. https://pubchem.ncbi.nlm.nih.gov.

[41] United States Pharmacopeia (2017). General Chapter. <621> Chromatography.

[42] Yang S., Hai F.I., Nghiem L.D., Price W.E., Roddick F., Moreira M.T., Magram S.F. (2013). Understanding the Factors Controlling the Removal of Trace Organic Contaminants by White-Rot Fungi and Their Lignin Modifying Enzymes: A Critical Review. Bioresour. Technol..

[43] Palli L., Castellet-Rovira F., Pérez-Trujillo M., Caniani D., Sarrà-Adroguer M., Gori R. (2017). Preliminary Evaluation of Pleurotus ostreatus for the Removal of Selected Pharmaceuticals from Hospital Wastewater. Biotechnol. Prog..

[44] Esterhuizen-Londt M., Hendel A.-L., Pflugmacher S. (2017). Mycoremediation of Diclofenac Using Mucor hiemalis. Toxicol. Environ. Chem..

[45] Lishman L., Smyth S.A., Sarafin K., Kleywegt S., Toito J., Peart T., Lee B., Servos M., Beland M., Seto P. (2006). Occurrence and Reductions of Pharmaceuticals and Personal Care Products and Estrogens by Municipal Wastewater Treatment Plants in Ontario, Canada. Sci. Total Environ..

[46] Celiz M.D., Tso J., Aga D.S. (2009). Pharmaceutical metabolites in the environment: Analytical challenges and ecological risks. Environ. Toxicol. Chem..

[47] Brown A.K., Wong C.S. (2015). Current Trends in Environmental Analysis of Human Metabolite Conjugates of Pharmaceuticals. Trends Environ. Anal. Chem..

[48] Brown A.K., Wong C.S. (2018). Distribution and Fate of Pharmaceuticals and Their Metabolite Conjugates in a Municipal Wastewater Treatment Plant. Water Res..

[49] Hai F.I., Yamamoto K., Nakajima F., Fukushi K., Nghiem L.D., Price W.E., Jin B. (2013). Degradation of azo dye acid orange 7 in a membrane bioreactor by pellets and attached growth of Coriolus versicolour. Bioresour. Technol..

[50] Margot J., Bennati-Granier C., Maillard J., Blánquez P., Barry D.A., Holliger C. (2013). Bacterial versus fungal laccase: Potential for micropollutant degradation. AMB Express.

[51] Chandel N., Ahuja V., Gurav R., Kumar V., Tyagi V.K., Pugazhendhi A., Kumar G., Kumar D., Yang Y.-H., Bhatia S.K. (2022). Progress in Microalgal Mediated Bioremediation Systems for the Removal of Antibiotics and Pharmaceuticals from Wastewater. Sci. Total Environ..

[52] Mojiri A., Zhou J.L., Ratnaweera H., Rezania S., Nazari V.M. (2022). Pharmaceuticals and Personal Care Products in Aquatic Environments and Their Removal by Algae-Based Systems. Chemosphere.

[53] Hejna M., Kapuścińska D., Aksmann A. (2022). Pharmaceuticals in the Aquatic Environment: A Review on Eco-Toxicology and the Remediation Potential of Algae. Int. J. Environ. Res. Public Health.

